# CoPc and CoPcF_16_ on gold: Site-specific charge-transfer processes

**DOI:** 10.3762/bjnano.5.61

**Published:** 2014-04-25

**Authors:** Fotini Petraki, Heiko Peisert, Johannes Uihlein, Umut Aygül, Thomas Chassé

**Affiliations:** 1 Institute of Physical and Theoretical Chemistry, University of Tübingen, Auf der Morgenstelle 18, 72076 Tübingen, Germany

**Keywords:** Auger parameter, charge transfer, interfaces, organic semiconductors, photoemission, phthalocyanines, polarization screening

## Abstract

Interface properties of cobalt(II) phthalocyanine (CoPc) and cobalt(II) hexadecafluoro-phthalocyanine (CoPcF_16_) to gold are investigated by photo-excited electron spectroscopies (X-ray photoemission spectroscopy (XPS), ultraviolet photoemission spectroscopy (UPS) and X-ray excited Auger electron spectroscopy (XAES)). It is shown that a bidirectional charge transfer determines the interface energetics for CoPc and CoPcF_16_ on Au. Combined XPS and XAES measurements allow for the separation of chemical shifts based on different local charges at the considered atom caused by polarization effects. This facilitates a detailed discussion of energetic shifts of core level spectra. The data allow the discussion of site-specific charge-transfer processes.

## Introduction

In order to develop and improve the performance of organic-based electronic devices an extended and comprehensive understanding of the basic physics appearing at the interface between organic and metallic materials is required. Molecules from the family of metal phthalocyanines have already been extensively applied to numerous molecular devices. In particular, opto-electronic devices such as light-emitting diodes, field-effect transistors, solar cells, and spintronic devices have been in the focus of research [1–4]. For several transition metal phthalocyanine (TMPc) layers on noble metal surfaces (e.g., Au and Ag) a charge transfer toward the central metal atom has been reported previously [5–13]. This affects the electronic and magnetic properties of the organic–metal interface and thus the performance of the molecular device. In the case of CoPc the open shell structure of Co can be easily affected by the presence of free electrons donated from a metallic substrate (e.g., Au, Ag) [6–7]. A complete description of the electronic situation and the energy level alignment at these interfaces must not only consider the observed metal-to-molecule charge transfer, but also other processes such as an adsorption-induced geometric distortion of the molecules, a possible molecule-to-metal back transfer, or a combination of these two [13–14].

The fluorination of phthalocyanines represents an ideal route for the tuning of the ionization potential (IP), a basic electronic parameter which can significantly affect the interface dipole and thus the energy level alignment [15–16]. In the context of applications, perfluorinated counterparts of Pcs have demonstrated a high performance and stability in air and are used as n-type channels in electronic devices [17–18]. It is interesting to see whether and how the fluorination of the molecules affects possible charge-transfer (or back-transfer) processes occurring at interfaces to metallic substrates. For the perfluorinated phthalocyanine ZnPcF_16_ on gold no evidence for a charge transfer between the central metal atom of the molecule and the substrate was observed, even if a charge-transfer screening of the Zn LMM Auger final state evidences an overlap of the wave functions of Zn with those of the Au substrate [19]. On the other hand, for perfluorinated CoPcF_16_ on Au(100) or Ag(111) an adsorption induced charge transfer including the central metal atom of the Pc is reported, pointing to a more complex interaction mechanism [13,20]. The aim of the present work is a more comprehensive study of the interfacial charge transfer between CoPc or CoPcF_16_ and metals by using core level X-ray photoemission spectroscopy (XPS), X-ray excited Auger electron spectroscopy (XAES), valence band ultraviolet photoemission spectroscopy (UPS) as well as X-ray absorption spectroscopy (XAS). Combined XPS and XAES measurements can be employed as a tool to study the contribution of the polarization energy to chemical shifts at interfaces. XAS gives valuable information about the unoccupied electronic structure and the hybridization of molecular levels at the interface (see, e.g., [21]).

## Results and Discussion

### Charge transfer to the central metal atom of the Pc

**Figure 1:**
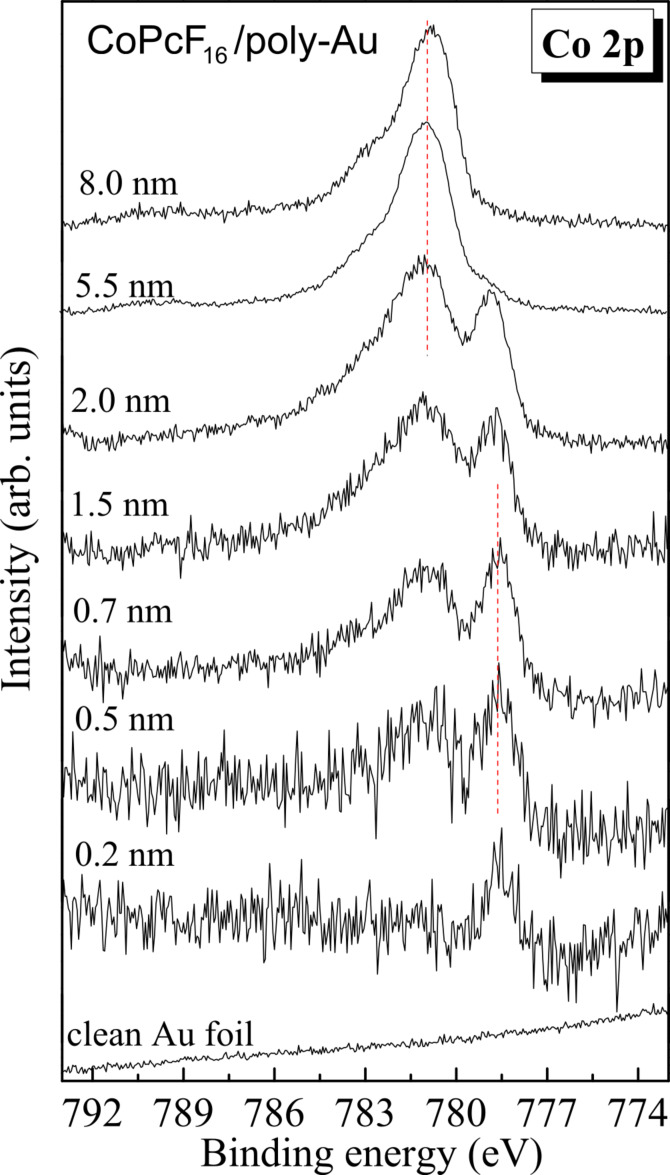
CoPcF_16_ on polycrystalline (poly-) Au: Co 2p core-level photoemission spectra (XPS, Al Kα) with increasing CoPcF_16_ film thickness on gold foil.

First we discuss Co 2p_3/2_ XPS core level spectra of CoPcF_16_ on polycrystalline Au as a function of the film thickness (Figure 1). The spectra on single crystalline Au(100) are very similar (see Supporting Information File 1). A distinct difference of the shape of the line is observed going from the interface to the bulk. A new peak appears at the low binding energy side at low coverage, the energy shift of which is about 2.3 eV with respect to the peaks for thicker, more bulk-like films. In addition, the shape of the spectrum changes for coverage in the monolayer range. In general, the shape of the Co 2p spectrum is determined by satellite features at higher binding energies arising from multiplet splitting due to the interaction of unpaired electrons in the photoemission final state. Therefore, a change of the spectral shape can be attributed to (i) an electron transfer from the metal surface, leading to a reduction of the Co(II) ion to Co(I), (ii) by a redistribution of the d-electrons, or both (i) and (ii). Both the appearance of an interface component and the change of the satellite structure at the interface were previously observed for the CoPc and related compounds on several metal surfaces [6–7,12,22]. Both phenomena reveal a change of the electronic configuration of the Co atoms at the interface by charge-transfer mechanisms. Taking into account a comparison of, e.g., CoPc on Au(100) and Ag(111), the detailed electronic situation might depend on the substrate under consideration [7]. In all cases the results indicate that an electron is transferred from the substrate to the Co atom of CoPc or CoPcF_16_.

### Polarization effects at the interface

Generally, the underlying reasons for energetic shifts in photoemission are complex. It is important to distinguish between a variation of the local charge at the considered atom and a different ability for a polarization screening of the environment. Combined X-ray photoemission spectroscopy (XPS) and X-ray excited Auger electron spectroscopy (XAES) can be used as a tool to study the screening mechanism of holes at organic interfaces [19,23–25]. The different final states in XPS (one hole) and XAES (two holes) cause different binding energy (*E*_B_) shifts. Frequently, for the analysis of these shifts the change of the modified Auger parameter α’ is monitored according to Δα’ = Δ*E*_B_(XPS) + Δ*E*_kin_(XAES) (*E*_kin_ corresponds to the kinetic energy). On the other hand, the modified Auger parameter α’ is correlated to the dynamical or one-hole relaxation energy *R*_D_ (Δα´≈ 2·Δ*R*_D_) and thus to the electronic polarization P. We note, however, that the discovery is hindered for the central metal atom of the TMPcs due to (i) an extra-molecular charge transfer within the time scale of the Auger-process or (ii) the change of the multiplet structure of the spectrum caused by charge-transfer processes in the initial state [5,19]. On the other hand, in case of fluorinated Pcs the absence of a local charge transfer process at the fluorine atom allows the estimation of the polarization screening via the corresponding Auger parameter [19].

**Figure 2:**
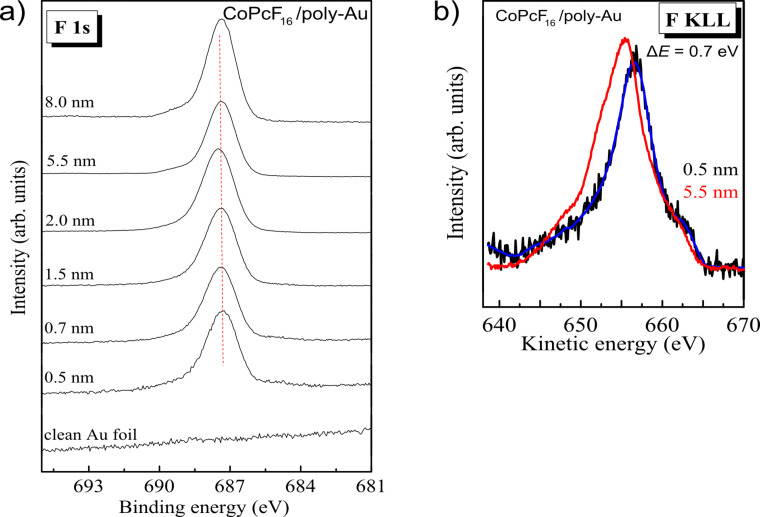
CoPcF_16_ on polycrystalline (poly-) Au: (a) F 1s core level spectra and (b) F KLL Auger spectra during the interface formation.

**Figure 3:**
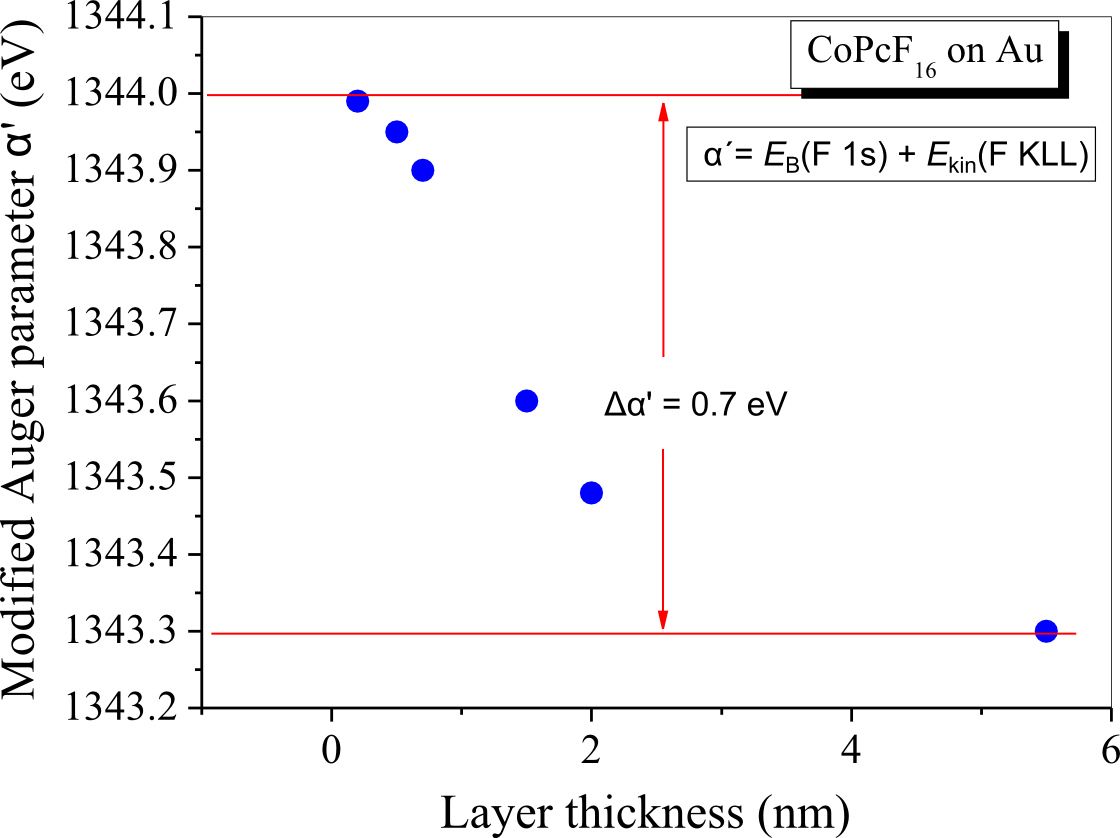
CoPcF_16_ on polycrystalline Au: Modified Auger parameter α’ for fluorine.

In Figure 2 we discuss F 1s core level spectra (Figure 2a) and F KLL Auger spectra (Figure 2b) for different film thicknesses of CoPcF_16_ on Au foil. Whereas the F 1s XPS core level spectra in Figure 2a show almost no change in the spectral shape and energetic position (+/− 0.1 eV) with increasing film thickness, the corresponding Auger spectra (Figure 2b) exhibit a significant energetic shift of about 0.7 eV toward lower kinetic energies. This points to the presence of polarization effects at the interface even if the photoemission spectrum shows no change. For a more detailed analysis the modified Auger parameter α΄ for fluorine (α΄= *E*_B_(F 1s) + *E*_K_(F KLL)) during the formation of the interface is plotted in Figure 3. Up to 5.5 nm a change of α΄of about 0.7 eV (+/− 0.2 eV) occurs, which corresponds to Δ*R*_D_ = 0.35 eV (+/− 0.1 eV). According to a previous study, where a dielectric continuum model for ZnPcF_16_ was applied [19], such values of the relaxation energy are reasonable for the first few organic layers and can be understood by polarization screening [26]. Thus, a small shift (0.3–0.4 eV) toward lower binding energies might be expected for all core levels at the interface compared to the bulk value. The question therefore arises why an energetic shift of F 1s to lower binding energies at the interface to Au is hardly observable.

### Bidirectional charge transfer

**Figure 4:**
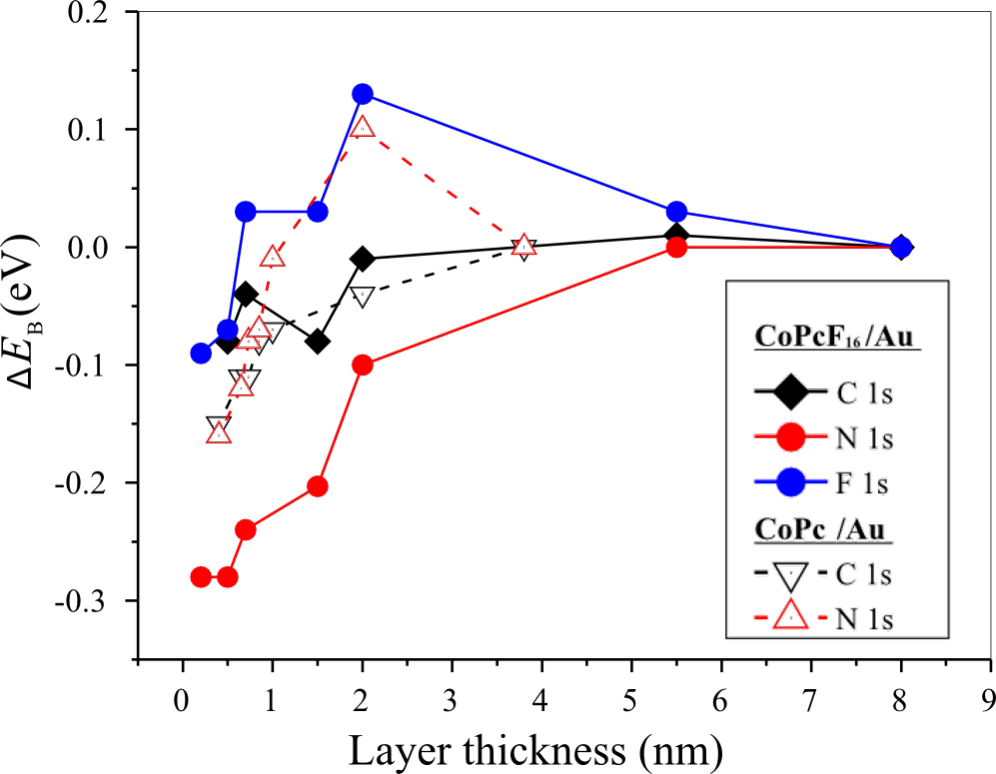
Comparison of energetic core level shifts as a function of the organic film thickness for CoPcF_16_ and CoPc on polycrystalline Au.

In order to understand the polarization and charge-transfer processes for the CoPcF_16_ macrocycle at the interface to poly-Au in more detail, we analyzed the energetic shifts of all core level lines of F 1s, N 1s and C 1s in Figure 4. The data were compared to CoPc/Au. Generally, only small changes of less than 0.4 eV could be observed for a CoPcF_16_ coverage of up to 2 nm. This might be expected for a polarization screening at the interface. On the other hand, it is visible from this figure that the core level spectra of different atoms directly at the interface (and up to 2 nm of film thickness) are shifted by different amounts. A shift of about 0.2 eV to a higher binding energy (*E*_B_) from the interface (about monolayer) to 2 nm thickness may be observed for the electronegative fluorine and nitrogen in CoPcF_16_, while the carbon peak shift <0.1 eV is hardly recognizable. With increasing film thickness the N 1s shift increases to ≈0.3 eV, while both F 1s and C 1s exhibit total shifts of only ≈0.1 eV. This unequal behavior can be explained by (i) a different polarization screening for each atom at the interface or (ii) a superposition of chemical shifts and polarization screening at the interface. A different polarization screening may arise from a bending of the molecule at the interface accompanied with different distances for each atom from the substrate surface as suggested for CuPcF_16_ on Cu(111) and Ag(111) [27]. However, the increase of the distance of fluorine on these systems is less than 0.03 nm and thus the expected change of the polarization screening is rather small (less than 0.08 eV [19]). Also, we rule out radiation damage since the shape of the Carbon 1s XPS spectrum is independent on the radiation exposure and film thickness. Thus, we conclude that chemical shifts toward higher binding energies compensate (partly) the expected (physical) shifts toward lower binding energies due to polarization screening. The chemical shift to higher binding energies, most clearly visible for F and C, implies that the phthalocyanine macrocycle is positively charged compared to molecules in the bulk. This means, while we observe an electron transfer to Co of CoPcF_16_, an opposite charge transfer is observed between the macrocycle of the molecule and the substrate, i.e., the charge transfer is bidirectional as observed for related systems [12,14,28]. Moreover, from the different energetic shifts we can conclude that the negative charge resides in the inner part of the molecule, whereas positive charges are observed primarily in the outer part – in the case of fluorine, the atoms are “less negatively” charged compared to the bulk.

**Figure 5:**
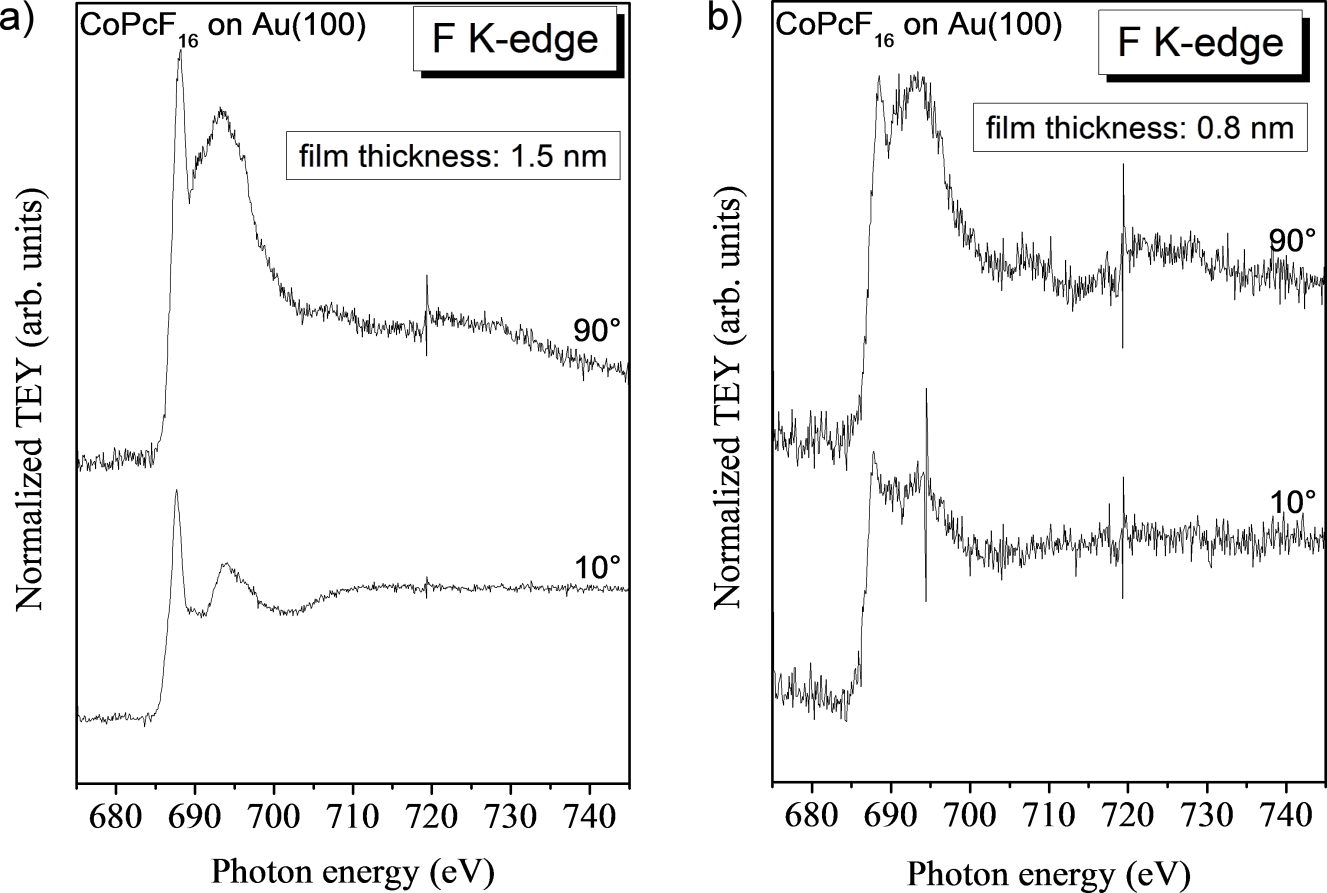
Angle dependent F K-edge XAS spectra for (a) 1.5 nm CoPcF_16_ on Au(100) and (b) 0.8 nm CoPcF_16_on Au(100). The different peak shape in both cases indicates a different electron distribution at the interface.

For a strongly related system, namely CoPcF_16_ on Au(100), X-ray absorption spectroscopy measurements at the fluorine K-edge show that the electron density can change at the fluorine site. Since XAS probes transitions from occupied into unoccupied valence states information about the unoccupied electronic structure is accessible. In Figure 5 we compare F K-edge spectra for two different film thicknesses acquired at a grazing and at a normal incidence of radiation. From N K absorption spectra (data not shown) we conclude that the molecules are flat lying on the substrate surface, thereby being in good agreement with related phthalocyanine films on single crystalline metal surfaces (see, e.g., [29]). Consequently, we observe transitions in the molecular plane at a normal incidence and transitions perpendicular to the molecular plane are probed at a grazing incidence (out of plane). The assignment of the two prominent features at photon energies of about 688 and 693 eV is complicated. It was reported that resonances in π* orbitals overlap in energy with σ* resonances at only slightly higher energies but with a much larger intensity. This results in a reversed linear dichroism of F K-edge XAS spectra compared to N or C K-edge spectra [30]. The presence of angular dependent π* and σ* transitions indicates that fluorine atoms participate in the conjugated π system. Comparing spectra from the thick film in Figure 5a to spectra at a lower coverage of about 0.8 nm (approximately 2 monolayers, Figure 5b), it is clearly visible that the shape of the spectra is changed, indicating a different electron distribution at the fluorine atom at the interface.

**Figure 6:**
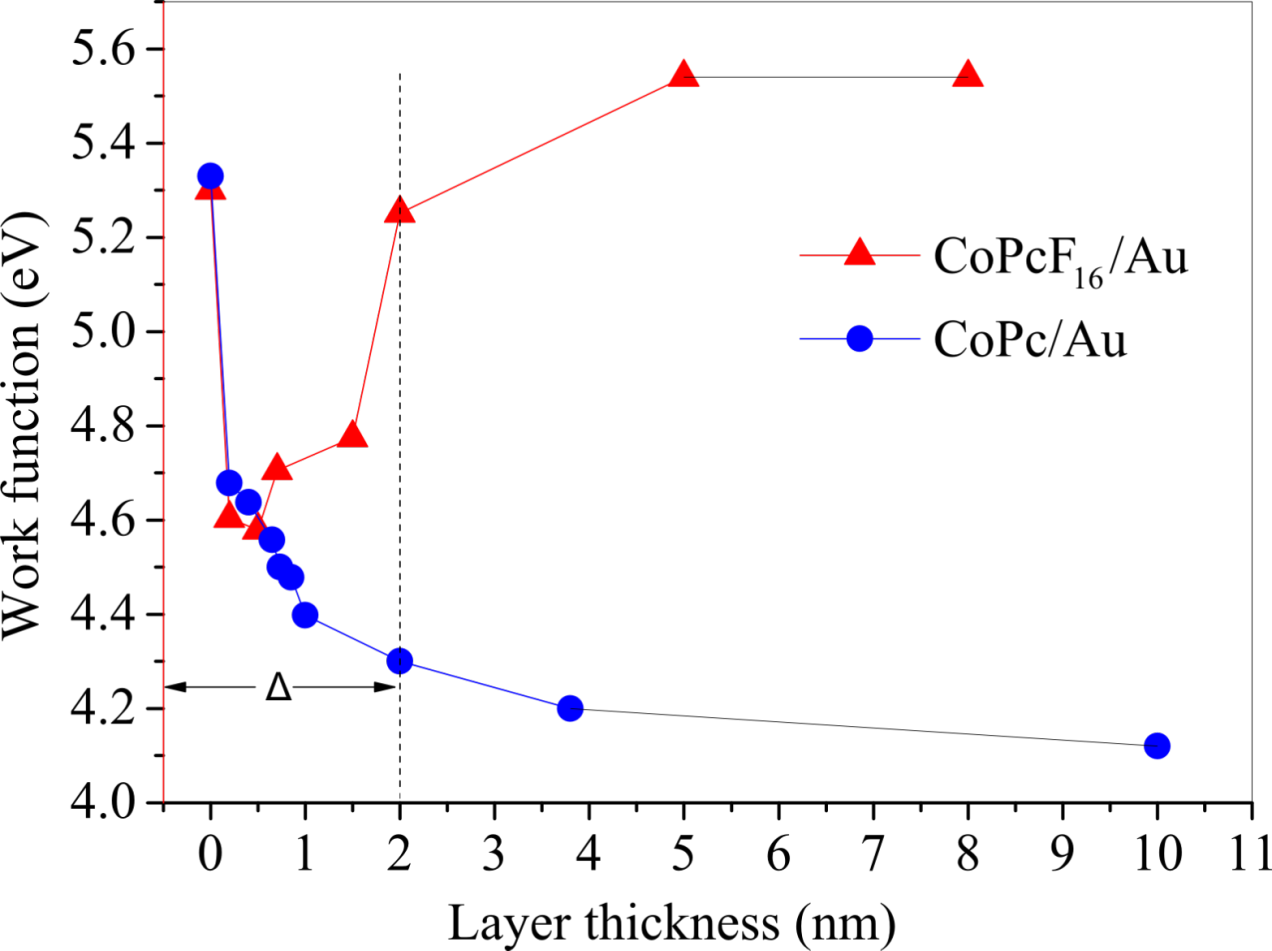
Energetic shift of the work function determined from the high binding energy cut-off in UPS spectra as a function of the film thickness for CoPcF_16_ and CoPc on polycrystalline Au. The observed shift is attributed to the formation of an interfacial dipole Δ.

The results show that only the consideration of several charge-transfer processes resulting in an inhomogeneous distribution of transferred charges may sufficiently explain “macroscopic” electronic interface properties such as the size of dipoles. The formation of interface dipoles can be monitored by work function (Φ) measurements, where a change of Φ at the interface indicates the formation of an interface dipole. Figure 6 displays the development of the work function determined by using UPS as a function of the CoPcF_16_ thickness on gold. The corresponding values for the CoPc–Au interface studied previously [6] were added for comparison. A strong decrease of the work function occurs for the very first steps of the deposition on both interfaces, relating to coverage in the monolayer range. Such potential drops at the interface can be attributed to a modification of the work function of gold upon adsorption of molecules due to the push back of the electron cloud of the metallic substrate. The extent of the changes of the work function caused by an adsorption of molecules (often also called Pauli repulsion or pillow effect) is considered a controversial issue, but for several systems values in the order of ≈0.3 eV are found [31–34]. Therefore, it seems that further processes contribute to the potential change at the interface, including charge transfer across the interface and intramolecular charge transfer. These effects seem to be remarkably similar for both types of molecules in the initial stage of adsorption. For thicker layers beyond ≈1 nm the behavior of the work function between the two interfaces changes, and with the development of bulk-like PC films beyond ≈2 nm (Δ) approaches to saturation levels. In the case of CoPc a total decrease of the work function of approximately 1.1 eV leads to a work function equal to 4.20 ± 0.1 eV for a thick (about 4 nm) CoPc layer. In case of CoPcF_16_ after the monolayer coverage there is a tendency of the work function toward higher values, reaching a plateau at about 5.50 ± 0.1 eV. Recently calculated energy level diagrams predict molecular HOMO (LUMO) energies of about −5.3 eV (−3.1 eV) and −6.7 eV (−4.5 eV) for CoPc and CoPcF_16_, respectively [35]. The difference of, e.g., calculated HOMO energies of 1.4 eV fits well to the observed work function difference of 1.3 eV. This implies that the related energy shifts with respect to the Au Fermi level are driven by the adjustment of chemical potentials to equilibrium at the interface between the metal substrate and the formed phthalocyanine film. A downward (upward) energy shift arises at the interface due to the location of the *E*_F_ or the midgap energy for free molecules above (below) the Fermi level of the metal substrate [35]. Therefore, a corresponding negative (positive) vacuum level shift strongly sustains an electron flow from the molecule (substrate) to the substrate (molecule) for CoPc/Au (CoPcF_16_/Au). We conclude that there is a positive total charge on CoPc at the established gold interface, whereas there only is a rather weak negative charge on the organic side in the case of CoPcF_16_ on Au. However, taking into account the charge transfer from gold to Co (see above), the macrocycle of the Pc must be positively charged in both cases: For CoPcF_16_ the dipoles related to positive (macrocycle) and negative charge transfer (Co) virtually compensate, whereas in the case of CoPc the positive charge transfer even exceeds the negative charge transfer. This is also reflected in the shifts of the binding energy of the respective core levels in Figure 4. In general, the observed binding energy shifts for C 1s and F 1s at the interface are lower than expected hinting at a positively charged macrocycle in both cases (see above). The binding energy of N 1s for CoPcF_16_ is decreased by about 0.3 eV matching the expectations of the polarization screening. In the case of CoPc the shift of N 1s is partly overwhelmed by a chemical shift due to a positive charge transfer. This points to a higher positive charge at the N sites in the case of CoPc.

The development of the work function as a function of the film thickness for CoPc and CoPcF_16_ on Au as well as the energy level alignment is in remarkable agreement to other Pcs such as CuPc/CuPcF_16_ [16] or ZnPc/ZnPcF_16_ [19] even if the charge transfer to the respective central metal atom is clearly different (no charge transfer was observed for ZnPc/ZnPcF_16_ [19]). It seems that the charge on the macrocycle of the Pc can compensate possible local charge transfer processes between the central metal atom of the Pc and the substrate adjusting the interface energetics.

## Conclusion

Combined photoemission spectroscopy and X-ray excited Auger electron spectroscopy was used as a tool to study the screening mechanism of holes at organic interfaces. This allows for the discrimination between chemical shifts due to a different local charge at the considered atom and polarization effects, thereby facilitating a detailed discussion of the energetic shifts of core level spectra. The Co 2p XPS spectral change reveals a strong charge donation from the underlying metal to the Co-atoms of the phthalocyanine. On the other hand, binding energy shifts of core level spectra representative for the Pc macrocycle point to an opposite charge transfer. The detailed analysis indicates that the positive charge is differently distributed over the Pc macrocycle. Together with UPS data we have shown that a bidirectional charge transfer determines the interface energetics for CoPc and CoPcF_16_ on Au.

## Experimental

X-ray photoemission spectroscopy (XPS), ultraviolet photoemission spectroscopy (UPS) and X-ray excited Auger electron spectroscopy (XAES) measurements were performed by using a multichamber UHV-system (base pressure 2 × 10^−10^ mbar), equipped with a Phoibos 150 cylindrical hemispherical analyzer (SPECS), a monochromatic Al Kα source, and a high-flux He discharge lamp (UVS 300, SPECS). The energetic resolution determined from the width of the Fermi edge for XPS and UPS was about 400 meV and 100 meV, respectively. The binding energy (*E*_B_) scale of the spectra was calibrated to reproduce the *E*_B_ of Au 4f_7/2_ (84.0 eV), Ag 3d_5/2_ (368.3 eV) and Cu 2p_3/2_ (932.5 eV). The cleanliness of the Au substrate was checked by XPS. Thin films of CoPcF_16_ (purchased from Aldrich) were thermally evaporated on the substrate in an ultra-high vacuum (base pressure <1 × 10^−8^ mbar) from a temperature-controlled evaporation cell. The thickness of the organic films ranged from sub-monolayer to about 100 Å and was determined by the attenuation of the intensity of the Au 4f substrate peaks in photoemission.

X-ray absorption (XAS) measurements were carried out at the third generation synchrotron radiation source BESSY II (Berlin) by using the Optics-beamline and the end-station SurICat. XAS spectra were acquired in total-electron yield (TEY) mode.

## Supporting Information

File 1 Co 2p core-level photoemission spectra.
