# Supplementary material for: CoPc and CoPcF16 on gold: Site-specific charge-transfer processes
Source: Beilstein J Nanotechnol. 2014 Apr 25;5:524–31. doi: 10.3762/bjnano.5.61 (PMC4077293; doi:10.3762/bjnano.5.61)
Supplement: File 1 — Co 2p core-level photoemission spectra. [file Beilstein_J_Nanotechnol-05-524-s001.pdf]

# Supporting Information
# for
# CoPc and $CoPcF_{16}$ on gold: Site-specific charge-transfer processes

Fotini Petraki, Heiko Peisert*, Johannes Uihlein, Umut Aygül and Thomas Chassé

Address: Institute of Physical and Theoretical Chemistry, University of Tübingen, Auf der Morgenstelle 18, 72076 Tübingen, Germany

Email: Heiko Peisert - heiko.peisert@uni-tuebingen.de

* Corresponding author

## Co 2p core-level photoemission spectra

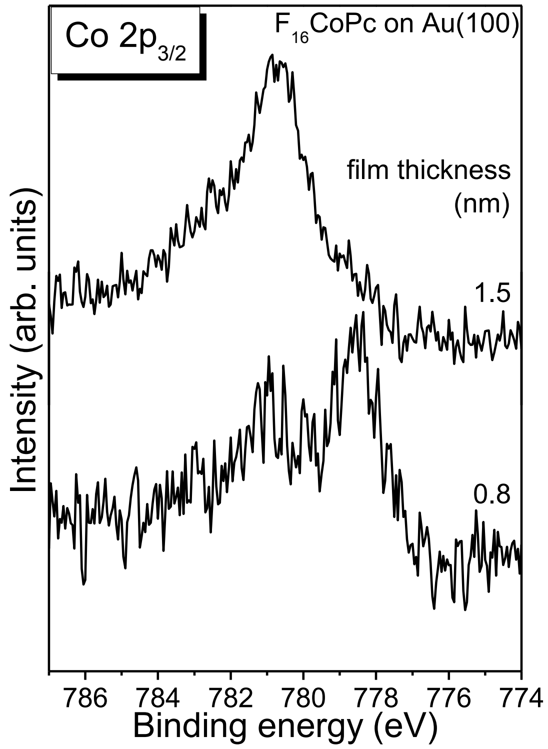


**Figure S1:** $CoPcF_{16}$/Au(100): Co 2p core-level photoemission spectra taken at an excitation energy of 900 eV as a function of the $CoPcF_{16}$ film thickness.

Similar to $CoPcF_{16}$ on Au foil, the data show an additional feature at low coverage (0.8 nm, 1–2 monolayers), which can be attributed to an interfacial charge transfer from the substrate to the Co ion of CoPc.
